# Multiple biomarker models for improved risk estimation of specific cardiovascular diseases related to metabolic syndrome: a cross-sectional study

**DOI:** 10.1186/s12963-015-0041-5

**Published:** 2015-03-14

**Authors:** Evan Coffman, Jennifer Richmond-Bryant

**Affiliations:** National Center for Environmental Assessment, U.S. Environmental Protection Agency, Oak Ridge Institute for Science and Education stationed at the Environmental Media Assessment Group, Mail Drop B243-01, Research Triangle Park, NC 27711 USA; Environmental Media Assessment Group, National Center for Environmental Assessment, U.S. Environmental Protection Agency, Mail Drop B243-01, Research Triangle Park, NC 27711 USA

**Keywords:** Cardiovascular disease, Biomarkers, Metabolic syndrome, Joint associations, NHANES

## Abstract

**Background:**

Metabolic syndrome (MetS) is the co-occurrence of several conditions that increase risk of chronic disease and mortality. Multivariate models for calculating risk of MetS-related diseases based on combinations of biomarkers are promising for future risk estimation if based on large population samples. Given biomarkers’ nonspecificity and commonality in predicting diseases, we hypothesized that unique combinations of the same clinical diagnostic criteria can be used in different multivariate models to develop more accurate individual and cumulative risk estimates for specific MetS-related diseases.

**Methods:**

We utilized adult biomarker and cardiovascular disease (CVD) data from the National Health and Nutrition Examination Survey as part of a cross-sectional analysis. Serum C-reactive protein (CRP), glycohemoglobin, triglycerides, high-density lipoprotein cholesterol, low-density lipoprotein cholesterol, total cholesterol, fasting glucose, and apolipoprotein-B were modeled. CVDs included congestive heart failure, coronary heart disease, angina, myocardial infarction, and stroke. Decile analysis for disease prevalence in each biomarker group and multivariate logistic regression for estimation of odds ratios were employed to measure the joint association between multiple biomarkers and CVD diagnoses.

**Results:**

Of the biomarkers considered, glycohemoglobin, triglycerides, and CRP were consistently associated with the CVD outcomes of interest in decile analysis and were selected for the final models. Associations were overestimated when using single-marker models in comparison with full models; individual odds ratios decreased an average of 16.4% from the single-biomarker models to the joint association models for CRP, 6.6% for triglycerides, and 1.4% for glycohemoglobin. However, joint associations were stronger than any single-marker estimate. Additionally, reduced models produced unique combinations of biomarkers for specific CVD outcomes.

**Conclusion:**

The reduced joint association modeling results suggest that unique combinations of biomarkers with their related measure of association can be used to produce more accurate cumulative risk estimates for each CVD. Additionally, our results indicate that the use of multiple biomarkers in a single multivariate model may provide increased accuracy of individual biomarker association estimates by controlling for statistical artifacts and spurious relationships due to co-biomarker confounding.

**Electronic supplementary material:**

The online version of this article (doi:10.1186/s12963-015-0041-5) contains supplementary material, which is available to authorized users.

## Background

Metabolic syndrome (MetS) is the co-occurrence of several conditions to present elevated risk of cardiovascular disease (CVD), as well as other diseases, including type II diabetes, fatty liver, and all-cause mortality [1-5]. The National Cholesterol Education Program’s Adult Treatment Panel III (ATP III) defined six criteria for MetS, including central obesity, dyslipidemia, elevated blood pressure, insulin resistance/glucose intolerance, proinflammatory state, and prothrombotic state [3]. MetS is often indicated clinically by biomarkers including waist circumference, cholesterol, triglycerides, blood pressure, fasting glucose, and C-reactive protein (CRP). The ATP III recommended clinical criteria to diagnose MetS [3]:

- Waist circumference ≥ 102 cm (men), ≥ 88 cm (women);
- Triglycerides ≥ 150 mg/dL;
- High-density lipoprotein cholesterol (HDL-C) ≤ 40 (men), ≤ 50 (women);
- Systolic blood pressure (SBP) ≥ 130 and/or diastolic blood pressure ≥ 85; and
- Fasting glucose ≥ 110 mg/dL.

Clinical criteria are important risk factors for CVDs associated with MetS, but they are nonspecific. Ridker et al. used data from the Women’s Health Study to examine potential differences in risk factors between women with and without nonspecific CVD [6]. They observed significant elevations among women with CVD for several biomarkers, including body mass index (BMI), CRP, total cholesterol (TC), low-density lipoprotein cholesterol (LDL-C), and apolipoprotein-B (apoB). Ridker and Anand et al. also reviewed the literature for use of biomarkers to predict CVD and observed no evidence of an optimal biomarker [7]. However, multivariate models calculating risk of disease based on combinations of biomarkers have provided reasonable estimates and offer promising options for future risk estimation if based on large populations [8]. Recent large-scale studies where risk of CVD was estimated from combinations of biomarkers have included the Framingham Heart Study [9], the Systematic Coronary Risk Evaluation system by the European Society of Cardiology [10], and the Prospective Cardiovascular Münster Study [11]. Although biomarker models are not designed to predict causality of disease, such multibiomarker associations are useful for estimating risk.

Given the nonspecificity and overlapping of biomarkers in predicting disease, we hypothesize that unique combinations of biomarkers can be used in different multivariate models to develop more accurate risk estimates for specific MetS-related diseases by reducing statistical artifacts and spurious relationships due to co-biomarker confounding. Hence, the objective of this study is to test for unique models in which multiple MetS biomarkers are employed jointly to assess more accurately their association with specific CVDs for different age groups, obtain a cumulative perspective of their impact on CVD, and provide a basis for future development of predictive models. Such predictive models may be used in the future both for estimates of population risk and to approximate an individual’s risk based on her/his combination of biomarkers. Decile analysis and regression are used to test these relationships for the nationally representative National Health and Nutrition Examination Survey (NHANES) sample dataset. These analyses are presented for the purpose of estimating risk rather than to make a specific assessment of causality.

## Methods

### Study design

NHANES is comprised of interview (demographics, socioeconomic status, dietary habits, and medical history), examination (dental, medical, and physiological evaluation), and laboratory segments [12-17]. NHANES uses a complex, multistage, unequal probability of selection cluster design to provide a nationally representative sample of the non-institutionalized US civilian population. We conducted a cross-sectional study using data from six independent two-year cycles of publicly available NHANES data spanning 1999–2010. The NHANES protocol has been approved by the National Center for Health Statistics Institutional Review Board, and written informed consent was obtained from all participants. NHANES methodology and sample design have been previously detailed elsewhere [18,19].

### Study population

The study population consisted of adults ≥ 20 years who completed the laboratory component of the survey and answered questions about their history of CVD and related health outcomes. Within the target age group, *n* = 32,458 participants answered questions regarding their cardiovascular health. The subjects’ health data included SBP and variables describing if they were ever told by a health care professional that they had congestive heart failure (CHF), coronary heart disease (CHD), stroke, myocardial infarction (MI), or angina. Demographic data (poverty-income ratio, sex, age, race/ethnicity), smoking history, and BMI were also employed in the analysis.

### Biomarkers

The biomarkers tested included CRP, glycohemoglobin, plasma fasting glucose, ApoB, TC, LDL-C, HDL-C, and triglycerides. These biomarkers were included because they were obtained from blood samples during laboratory testing of NHANES participants. Fasting glucose, ApoB, LDL-C, and triglyceride serum levels were only measured in a subsample consisting of one-third of all persons 12 years and older in each NHANES cycle. The subsamples were nationally representative, and appropriate sample weights were applied to account for oversampling. Blood samples were drawn, stored, and analyzed according to specific protocols [20-25].

### Statistical analyses

Summary statistics were tabulated to describe the general population characteristics, biomarkers, and health outcomes. Appropriate stratum values, sampling units, and survey weights were applied so that nationwide inference can be drawn [26]. Likewise, sample weights were applied in all regression models.

Multivariate logistic regression was used to estimate odds ratios (ORs) and joint association ORs to measure the association between multiple biomarkers and CVDs [27]. For the purpose of this study, joint associations measure the odds of prevalent CVD respective to the simultaneous concentrations of each measured biomarker. Whereas an interaction would measure how an individual association between a CVD outcome and a biomarker concentration varies given the change in concentration of another biomarker, a joint association measures the aggregated impact on odds of disease for the selected markers by controlling for potential confounding and co-explanation of disease between the co-related variables. Biomarkers used in the final models were determined by examining significant changes in CVD prevalence across deciles of biomarker serum concentrations. Next, we removed highly correlated biomarkers. Although joint associations are robust to correlated variables [28], we were also interested in the individual associations of single biomarkers within the joint biomarker model, which can be impacted by correlation. We used log-transformation to account for highly skewed biomarkers in the models.

After the final biomarkers were determined, adjusted single biomarker regression models were calculated for each cardiovascular health outcome to serve as a level of comparison for the joint effect models. Base joint effect models for each CVD had one term for each log-transformed biomarker as an independent variable. By including multiple biomarkers, we simultaneously controlled for potential confounding and overlapping associations between biomarkers. We then built unique models for each CVD by removing nonsignificant biomarkers from the base model using iterative backwards elimination [29]. R^2^ values are often used to assess model fit and predictive power in linear regression but cannot be calculated directly for logistic regression models. Statistical software calculated pseudo-R^2^ values for logistic regression, but we decline to present them because they are not a measure of explained variability and could potentially mislead the reader [30,31]. In these joint association models, the log-transformed biomarker terms serve simultaneously as independent variables making up a portion of the overall joint association and control variables when examining the individual impact of a specific biomarker within the model. Because the biomarkers serve as control variables, we compared the reduced model single-biomarker OR estimates to the full model single-marker estimates to assess meaningful changes due to the removal of biomarker terms from the models. In addition to base and reduced models for the total study population, we stratified the base models by age to examine potential differences.

Joint association ORs were calculated by taking the exponentiated sum of the product of each log-transformed biomarker regression coefficient and that respective log-transformed biomarker’s interquartile range (IQR). Using log-transformed IQR increments provided standardization across biomarkers, making it easier to interpret the joint association OR. Standard errors used for calculating the joint association model confidence intervals (CIs) were determined using the covariance matrices for each individual biomarker estimate, as described in Winquist et al. and in Additional file 1 [27].

To control for potential confounders, we adjusted for sex, age, race/ethnicity, BMI, smoking history, SBP, and family income. We separated age into four groups: 20–34, 35–44, 45–60, and 60+. Race/ethnicity was categorized as: Mexican-American, white, black, and other. BMI was classified, according to NIH guidelines, as: underweight (<18.5 kg/m^2^), normal weight (18.5-25.0 kg/m^2^), overweight (25.0-30 kg/m^2^), and obese (>30 kg/m^2^). We divided subjects’ smoking status into: nonsmokers, former smokers, those who smoke some days, and every-day smokers. SBP measures were categorized according to American Heart Association classifications: normal (<120 mmHg), prehypertension (120–139 mmHg), Stage 1 hypertension (140–159 mmHg), and Stage 2 hypertension (>159 mmHg). Family income was measured as a ratio of income-to-poverty threshold and then divided into survey-weighted quartiles.

All analyses were conducted using SAS v9.3 (SAS Institute Inc., Cary, NC). SAS sampling and survey analysis procedures were used to implement NHANES stratum values, sampling units, and survey weights to account for unequal selection probability and the intentional oversampling of demographic groups as a part of the NHANES complex, multistage cluster design [26]. Survey weights were recalculated for the 10-year period before they were applied.

## Results

### Study population

The study population consisted of *n* = 32,458 subjects meeting the inclusion criteria. Sample size for the final regression models ranged from *n* = 2,119 to *n* = 28,348, depending on age-stratification and inclusion of certain biomarkers that were measured in a subsample of the study population. There were more females *n* = 16,936 than males *n* = 15,522, while whites were the most highly represented racial/ethnic group at just over 70% (Table 1). All study population statistics reflect application of survey weights.

**Table 1:** **Study population characteristics**, **US adults aged 20 and older**, **NHANES 1999**-**2010**

	**n (% within group** ^**a**^ **)**
**Overall**	32,458 (100.0)
**Sex**	
Male	15,522 (48. 0)
Female	16,936 (52.0)
**Age** **(years)**	$\left(\overline{\mathrm{x}}=46.5\right)$
20-34	8,576 (28.3)
35-44	5,483 (20.7)
45-60	7,649 (29.2)
60+	10,750 (21.8)
**Ethnicity**	
Mexican-American	6,552 (7.8)
White	15,993 (70.4)
Black	6,388 (11.2)
Other	3,525 (10.6)
**BMI** ^**b**^	
Underweight (0–18.5)	493 (1.9)
Normal (18.5-25)	8,656 (31.4)
Overweight (25–30)	10,491 (34.0)
Obese (30+)	10,349 (32.7)
**Smoking status**	
Nonsmoker	17,008 (51.7)
Former smoker	8,350 (24.7)
Some days	1,229 (3.7)
Every day	5,824 (19.9)
**Systolic blood pressure**	
Normal (<120 mmHg)	15,166 (52.5)
Prehypertension (120–139)	9,608 (32.1)
Stage 1 hypertension (140–159)	3,937 (10.8)

^a^Survey-weighted.

^b^BMI is only available for a smaller subset of participants who completed the mobile exam center exam.

### Serum biomarker concentrations

There were significant sex-related differences in survey-weighted means of each biomarker (Table 2). Geometric means were calculated for biomarkers with skewed distributions, resulting in a measure of central tendency that is less influenced by extreme outliers. Men had significantly higher geometric mean glycohemoglobin, fasting glucose, ApoB, and triglycerides levels than women, as well as higher mean LDL-C. Meanwhile, women had higher TC and HDL-C mean levels and CRP geometric mean levels than men. There were significant differences in all biomarkers among age, race/ethnicity, and BMI quartile groups, while disparities across smoking status and income-to-poverty quartile were present for most biomarkers.

**Table 2:** **Demographic**-**stratified survey**-**weighted laboratory biomarker means and 95**% **confidence intervals**

	**CRP** ^**a**^ **(mg**/**dL)**		**Glycohemoglobin** ^**a**^ **(%)**		**Fasting Glucose** ^**a**,**b**^ **(mg/dL)**		**ApoB** ^**a**,**b**^ **(mg/dL)**		**Total cholesterol (mg/dL)**		**HDL-C (mg/dL)**		**LDL-C** ^**b**^ **(mg/dL)**		**Triglycerides** ^**a,b**^ **(mg/dL)**	
	$\overline{\mathbf{x}}$	**95% CI**	$\overline{\mathbf{x}}$	**95% CI**	$\overline{\mathbf{x}}$	**95% CI**	$\overline{\mathbf{x}}$	**95% CI**	$\overline{\mathbf{x}}$	**95% CI**	$\overline{\mathbf{x}}$	**95% CI**	$\overline{\mathbf{x}}$	**95% CI**	$\overline{\mathbf{x}}$	**95% CI**
**Overall**	0.185	0.179, 0.190	5.46	5.44, 5.48	100.4	99.8, 100.9	91.6	90.4, 92.9	200.0	199.2, 200.8	52.7	52.4, 53.1	118.2	117.3, 119.1	116.8	115.0, 118.5
**Gender**																
Male	0.154	0.149, 0.159	5.49	5.47, 5.51	103.2	102.5, 103.9	93.5	92.3, 94.7	198.2	197.1, 199.2	47.1	46.8, 47.5	119.4	118.2, 120.6	125.4	122.7, 128.2
Female	0.219	0.211, 0.227	5.44	5.42, 5.46	97.8	97.1, 98.4	89.9	88.4, 91.4	201.7	200.7, 202.7	58.0	57.4, 58.5	117.0	115.8, 118.2	109.2	107.2, 111.3
**Age (years)**																
20-34	0.144	0.138, 0.150	5.16	5.14, 5.18	93.1	92.6, 93.7	84.5	83.1, 85.9	185.7	184.6, 186.8	51.4	50.9, 51.8	109.7	108.1, 111.2	101.4	98.6, 104.2
35-44	0.171	0.162, 0.180	5.34	5.31, 5.36	97.6	96.7, 98.6	91.9	89.6, 94.3	201.7	200.1, 203.3	51.7	51.1, 52.3	119.0	117.0, 120.9	115.2	111.0, 119.7
45-60	0.202	0.193, 0.211	5.61	5.58, 5.63	103.7	102.8, 104.7	97.8	95.9, 99.8	209.8	208.2, 211.4	53.3	52.8, 53.9	126.0	124.3, 127.7	125.3	122.5, 128.1
60+	0.242	0.234, 0.249	5.80	5.77, 5.83	108.6	107.7, 109.6	92.4	90.9, 93.9	203.3	202.1, 204.5	54.7	54.1, 55.3	117.9	116.5, 119.4	129.4	127.2, 131.6
**Ethnicity**																
Mexican-American	0.207	0.194, 0.220	5.53	5.50, 5.57	102.9	101.5, 104.3	94.9	93.3, 96.5	197.6	196.3, 199.0	49.7	49.1, 50.2	117.3	115.5, 119.2	126.5	122.2, 131.0
White	0.180	0.174, 0.187	5.41	5.39, 5.44	100.0	99.3, 100.6	91.7	90.2, 93.2	201.2	200.2, 202.2	52.9	52.4, 53.4	118.8	117.7, 119.9	119.6	117.5, 121.7
Black	0.229	0.217, 0.242	5.65	5.62, 5.68	100.2	98.9, 101.5	88.9	87.3, 90.5	193.7	192.4, 194.9	55.8	55.3, 56.4	115.6	114.0, 117.2	91.3	88.5, 94.1
Other	0.160	0.147, 0.173	5.55	5.51, 5.60	101.4	99.8, 103.0	91.5	88.8, 94.3	200.0	197.7, 202.2	50.9	50.1, 51.8	117.1	114.3, 119.9	120.6	116.1, 125.4
**BMI**																
Underweight (0–18.5)	0.066	0.057, 0.078	5.18	5.15, 5.22	91.4	90.1, 92.7	76.2	71.7, 80.9	181.3	177.4, 185.1	64.4	62.3, 66.4	99.4	94.6, 104.2	80.2	74.0, 86.8
Normal (18.5-25)	0.101	0.096, 0.105	5.27	5.25, 5.29	94.8	94.2, 95.3	84.4	83.1, 85.7	194.1	192.9, 195.3	59.5	58.9, 60.0	112.4	111.0, 113.8	93.5	91.6, 95.4
Overweight (25–30)	0.174	0.169, 0.180	5.43	5.41, 5.45	100.5	99.7, 101.2	95.3	93.9, 96.8	204.9	203.6, 206.2	51.5	51.1, 52.0	122.9	121.6, 124.3	124.5	121.8, 127.2
Obese (30+)	0.363	0.354, 0.373	5.69	5.66, 5.71	106.3	105.4, 107.2	95.7	93.9, 97.5	201.7	200.5, 202.9	47.0	46.6, 47.4	120.4	118.9, 121.8	138.2	134.8, 141.6
**Smoking status**																
Nonsmoker	0.171	0.165, 0.178	5.44	5.42, 5.46	99.4	98.8, 100.0	90.5	89.1, 91.8	198.7	197.8, 199.6	53.7	53.3, 54.1	117.3	116.2, 118.4	110.0	107.7, 112.3
Former smoker	0.196	0.188, 0.205	5.55	5.52, 5.57	103.7	102.7, 104.8	93.1	91.5, 94.8	202.7	201.2, 204.2	53.1	52.5, 53.7	119.4	117.6, 121.2	125.3	121.9, 128.7
Some days	0.166	0.153, 0.180	5.37	5.31, 5.44	99.6	96.9, 102.5	90.9	86.7, 95.3	199.3	195.7, 202.8	52.8	51.5, 54.1	114.6	109.6, 119.6	115.5	109.6, 121.7
Every day	0.211	0.203, 0.205	5.44	5.42, 5.46	98.7	97.9, 99.6	93.1	90.9, 95.4	200.1	198.6, 201.5	49.7	49.1, 50.4	119.4	117.7, 121.2	125.1	121.9, 128.4
**Income to poverty quartile** ^**c**^																
Quartile 1	0.212	0.203, 0.221	5.53	5.51, 5.55	101.3	100.5, 102.2	91.0	89.5, 92.5	199.1	197.9, 200.3	51.3	50.7, 51.9	117.3	115.8, 118.9	120.0	117.2, 122.8
Quartile 2	0.195	0.186, 0.205	5.50	5.47, 5.53	101.7	100.6, 102.8	92.5	90.8, 94.2	199.1	197.8, 200.4	52.3	51.8, 52.8	118.0	116.5, 119.6	118.6	115.7, 121.7
Quartile 3	0.179	0.171, 0.187	5.42	5.40, 5.45	99.0	98.2, 99.9	90.7	88.7, 92.6	199.9	198.5, 201.2	52.6	52.1, 53.1	117.9	116.2. 119.6	114.0	110.7, 117.5
Quartile 4	0.158	0.150, 0.166	5.39	5.37, 5.42	99.5	98.7, 100.3	92.3	90.4, 94.3	201.7	200.2, 203.3	54.7	54.0, 55.4	119.3	117.3, 121.3	114.8	111.6, 118.0
**Systolic blood pressure**																
Normal (<120 mmHg)	0.162	0.157, 0.167	5.35	5.33, 5.37	97.0	96.3, 97.6	88.9	87.6, 90.3	195.5	194.5, 196.2	53.4	53.0, 53.8	115.5	114.3, 116.7	107.8	105.7, 109.8
Prehypertension (120–139)	0.199	0.191, 0.207	5.53	5.50, 5.55	103.1	102.3, 104.0	93.9	92.3, 95.5	202.7	201.5, 204.0	51.0	50.6, 51.5	120.5	118.9, 122.0	125.9	122.6, 129.3
Stage 1 hypertension (140–159)	0.237	0.224, 0.252	5.68	5.64, 5.71	106.9	105.5, 108.4	99.5	93.9, 99.5	209.0	207.0, 210.9	53.4	52.6, 54.2	123.3	120.8, 125.7	131.8	127.6, 136.2
Stage 2 hypertension (>159)	0.274	0.257, 0.291	5.82	5.76, 5.87	108.6	106.0, 111.3	100	97.6, 104	212.1	209.6, 214.7	55.9	54.9, 56.9	122.9	119.7, 126.1	137.0	130.4, 143.8

^a^Geometric mean.

^b^Population subsample.

^c^Income to poverty quartile is a categorical measure of the ratio of family income to poverty threshold (Q1: 0.00-1.33 Q2: 1.34-2.72 Q3: 2.73-4.65 Q4: 4.65-5.00).

### CVD prevalence

Survey-weighted prevalence of CVD ranged from 2.35% (95% CI: 2.13, 2.56) for CHF to 3.45% (95% CI: 3.16, 3.75) for MI (Table 3). The overall prevalence of CVD, measured as subjects who experienced at least one measured outcome, was 8.64% (95% CI: 8.10, 9.18). Depending on the health outcome of interest, prevalence varied significantly relative to sex, age, race/ethnicity, BMI, smoking status, and income-to-poverty ratio quartile group.

**Table 3:** **Demographic-stratified cardiovascular outcome prevalence and 95% confidence intervals**

	**Congestive heart failure**			**Coronary heart disease**			**Angina**			**MI**			**Stroke**		
	***n*** **(case/total)**	**%** ^**a**^	**95% CI** ^**a**^	***n*** **(case/total)**	**%** ^**a**^	**95% CI** ^**a**^	***n*** **(case/total)**	**%** ^**a**^	**95% CI** ^**a**^	**n (case/total)**	**%** ^**a**^	**95% CI** ^**a**^	**n (case/total)**	**%** ^**a**^	**95% CI** ^**a**^
**Overall**	1,122/32,333	2.35	2.13, 2.56	1,414/32,279	3.40	3.13, 3.67	1,065/32,329	2.60	2.32, 2.87	1,511/32,389	3.45	3.16, 3.75	1,284/32,408	2.75	2.51, 2.66
**Gender**															
Male	619/15,449	2.58	2.30, 2.86	941/15,426	3.40	4.20, 5.08	574/15,456	2.86	2.48, 3.23	993/15,489	4.55	4.09, 5.01	635/15,495	2.42	2.17, 2.66
Female	503/16,884	2.13	1.86, 2.41	473/16,853	2.25	1.96, 2.54	491/16,873	2.35	2.03, 2.68	518/16,900	2.44	2.15, 2.73	649/16,913	3.06	2.70, 3.41
**Age**															
20-34	14/8,574	0.19	0.08, 0.31	14/8,573	0.15	0.06, 0.25	13/8,571	0.14	0.04, 0.24	26/8,573	0.34	0.17, 0.50	30/8,576	0.38	0.22, 0.54
35-44	37/5,476	0.55	0.34, 0.77	32/5,475	0.65	0.40, 0.90	42/5,474	0.76	0.49, 1.03	53/5,477	0.81	0.53, 1.09	49/5,478	0.92	0.59, 1.26
45-60	204/7,634	1.98	1.58, 2.38	245/7,622	3.00	2.50, 3.50	215/7,672	2.61	2.09, 3.13	284/7,640	3.32	2.78, 3.86	218/7,642	2.23	1.83, 2.63
60+	867/10,649	7.38	6.76, 8.01	1,123/10,609	10.9	10.1, 11.7	795/10,657	7.56	6.78, 8.35	1,148/10,599	10.2	9.44, 11.0	987/10,712	8.30	7.53, 9.06
**Ethnicity**															
Mexican-American	137/6,511	1.00	0.80, 1.21	180/6,511	1.53	1.25, 1.81	150/6,518	1.32	0.94, 1.69	175/6,535	1.40	1.13, 1.67	168/6,544	1.34	1.03, 1.66
White	648/15,941	2.47	2.20, 2.75	965/15,908	4.02	3.67, 4.36	694/15,945	2.99	2.64, 3.34	983/15,957	3.94	3.58, 4.30	722/15,960	2.88	2.57, 3.19
Black	250/6,369	3.00	2.54, 3.46	168/6,358	1.94	1.62, 2.76	132/6,363	1.61	1.30, 1.93	249/6,382	2.98	2.57, 3.39	303/6,385	3.64	3.21, 4.07
Other	87/3,512	1.80	1.30, 2.31	101/3,502	2.19	1.62, 2.76	89/3,503	1.94	1.39, 2.49	104/3,515	2.20	1.72, 2.68	91/3,519	2.00	1.43, 2.57
**BMI**															
Underweight (0-18.5)	9/491	1.04	0.32, 1.76	14/490	1.84	0.66, 3.02	8/489	1.22	0.01, 2.43	19/492	2.34	0.99, 3.70	11/492	2.74	0.84, 4.64
Normal (18.5-25)	183/8,630	1.34	1.10, 1.58	271/8,620	2.16	1.86, 2.46	190/8,634	1.71	1.35, 2.08	288/8,644	2.28	1.94, 2.61	243/8,646	1.84	1.52, 2.16
Overweight (25-30)	313/10,450	2.18	1.88, 2.48	468/10,434	3.73	3.26, 4.21	321/10,448	2.48	2.10, 2.86	459/10,471	3.41	2.97, 3.86	364/10,474	2.58	2.24, 2.92
Obese (30+)	424/10,313	3.20	2.74, 3.65	500/10,297	4.26	3.78, 4.74	422/10,309	3.59	3.12, 4.07	552/10,327	4.47	3.99, 4.96	425/10,335	3.27	2.83, 3.71
**Smoking status**															
Non-smoker	446/16,959	1.75	1.50, 2.00	529/16,937	2.30	2.03, 2.58	417/16,945	1.94	1.65, 2.23	504/16,973	2.14	1.88, 2.41	540/16,989	2.27	2.00, 2.54
Former smoker	484/8,295	4.03	3.53, 4.52	685/8,274	6.81	6.08, 7.53	482/8,303	4.51	3.99, 5.03	697/8,332	6.34	5.63, 7.05	486/8,332	3.72	3.29, 4.15
Some days	28/1,228	1.79	0.92, 2.66	29/1,225	1.81	0.88, 2.79	29/1,229	1.85	1.11, 2.59	39/1,229	2.16	1.28, 3.04	34/1,229	2.13	1.23, 3.02
Every day	161/5,805	1.92	1.53, 2.30	169/5,797	2.32	1.85, 2.79	135/5,806	2.07	1.53, 2.61	269/5,811	3.51	2.97, 4.06	221/5,811	2.91	2.40, 3.42
**Income to poverty quartile** ^**b**^															
Quartile 1	504/11,836	3.38	2.98, 3.78	536/11,816	3.59	3.15, 4.03	423/11,839	3.22	2.71, 3.73	665/11,864	4.44	3.91, 4.96	581/11,886	3.82	3.36, 4.27
Quartile 2	352/8,239	3.35	2.93, 3.76	401/8,220	4.07	3.62, 4.52	337/8,236	3.21	2.75, 3.66	441/8,258	4.31	3.73, 4.89	369/8,255	3.57	2.97, 4.17
Quartile 3	171/6,315	1.74	1.34, 2.14	236/6,303	2.86	2.39, 3.32	156/6,314	1.97	2.75, 3.66	217/6,319	2.60	2.19, 3.01	209/6,320	2.18	1.82, 2.55
Quartile 4	95/5,943	0.94	0.70, 1.19	241/5,940	3.10	2.60, 3.61	149/5,940	1.98	1.53, 2.43	188/5,948	2.44	2.01, 2.88	125/5,947	1.43	1.14, 1.73
**Systolic blood pressure**															
Normal (<120 mmHg)	523/16,832	2.09	1.85, 2.33	559/16,808	2.54	2.23, 2.85	438/16,819	2.13	1.87, 2.38	647/16,845	2.78	2.45, 3.10	521/16,853	2.13	1.87, 2.38
Prehypertens ion (120-139)	273/9,581	1.94	1.64, 2.24	412/9,554	3.30	2.84, 3.76	303/9,579	2.41	2.01, 2.81	426/9,592	3.35	2.87, 3.82	342/9,596	2.47	2.12, 2.82
Stage 1 hypertens ion (140-159)	199/3,905	3.83	3.10, 4.58	283/3,899	6.47	5.41, 7.53	206/3,909	4.55	3.67, 5.42	280/3,925	5.99	4.94, 7.05	221/3,929	4.57	3.85, 5.29
Stage 2 hypertens ion (>160)	127/2,015	5.03	3.99. 6.06	160/2,018	7.90	6.29, 9.51	118/2,022	5.42	4.10, 6.73	158/2,027	6.86	5.63, 8.08	200/2,030	8.56	6.84, 10.3

^a^Survey-weighted.

^b^Income to poverty quartile is a categorical measure of the ratio of family income to poverty threshold (Q1: 0.00-1.02 Q2: 1.03-2.12 Q3: 2.13-3.87 Q4: 3.88-5.00.

### Serum biomarker deciles and CVD prevalence

We calculated survey-weighted deciles of biomarker concentrations to examine unadjusted associations between biomarkers and CVD outcomes. Participants within the top deciles of CRP, glycohemoglobin, fasting glucose, and triglycerides had significantly higher prevalence of each measured outcome than subjects in the bottom decile (Table 4) and thus were considered for our joint association models. Among the four remaining biomarkers, there were significant negative associations of decile-level TC, LDL-C, and HDL-C with CVD. With the exception of a negative association with CHD, associations with ApoB were not significant.

**Table 4:** **Summary statistics and survey-weighted CVD prevalence by survey-weighted deciles of laboratory biomarkers**

	**Summary statistics**			**Congestive heart failure**			**Coronary heart disease**			**Angina**			**MI**			**Stroke**		
	**Range**	$\overline{\mathbf{x}}$ ^**a**^	**95% CI** ^**a**^	***n*** **(case/total)**	**%** ^**a**^	**95% CI** ^**a**^	***n*** **(case/total)**	**%** ^**a**^	**95% CI** ^**a**^	***n*** **(case/total)**	**%** ^**a**^	**95% CI** ^**a**^	***n*** **(case/total)**	**%** ^**a**^	**95% CI** ^**a**^	***n*** **(case/total)**	**%** ^**a**^	**95% CI** ^**a**^
**C-reactive Protein (mg/dL)**																		
All	0.01-29.6	0.417	0.405, 0.429	916/28,817	2.28	2.06, 2.51	1,229/28,778	3.43	3.15, 3.71	921/28,816	2.62	2.33, 2.91	1,296/28,869	3.44	3.15, 3.73	1,055/28,882	2.68	2.42, 2.94
D1	0.01-0.03	0.021	0.020, 0.021	21/2,373	0.73	0.30, 1.16	39/2,371	1.39	0.89, 1.90	32/2,377	1.34	0.77, 1.91	48/2,381	1.39	0.84, 1.95	38/2,381	0.93	0.50, 1.36
D10	0.97-29.6	2.044	1.979, 2.108	202/3,350	5.28	4.37, 6.18*	178/3,343	4.5	3.68, 5.33*	163/3,347	4.41	3.58, 5.24*	222/3,361	5.28	4.42, 6.14*	193/3,362	4.92	4.05, 5.80*
**Glycohemoglobin (%)**																		
All	2.00-18.8	5.51	5.49, 5.53	926/29,000	2.30	2.08, 2.53	1,242/28,958	3.44	3.15, 3.72	932/28,999	2.63	2.34, 2.92	1,313/29,051	3.45	3.16, 3.75	1,074/29,066	2.71	2.44, 2.98
D1	2.00-4.83	4.67	4.66, 4.68	21/2,225	0.89	0.42, 1.36	28/2,227	0.87	0.44, 1.29	16/2,227	0.75	0.34, 1.16	34/2,227	1.16	0.63, 1.68	38/2,227	1.44	0.80, 2.08
D10	6.07-18.8	7.34	7.30, 7.42	363/4,390	7.47	6.44, 8.49*	415/4,379	9.69	8.48, 10.91*	315/4,386	7.13	5.94, 8.32*	456/4,409	9.70	8.60, 10.80*	342/4,417	6.98	6.04, 7.93*
**Fasting glucose (mg/dL)**																		
All	38.0-587	102.7	102.0, 103.4	377/12,929	2.18	1.87, 2.48	528/12,915	3.38	3.01, 3.74	401/12.920	2.60	2.21, 2.98	578/12,957	3.48	3.08, 3.88	457/12,962	2.70	2.33, 3.07
D1	38.0-84.9	80.4	80.0, 80.7	23/1,272	1.30	0.70, 1.91	23/1,269	1.7	0.77, 2.62	17/1,271	1.24	0.49, 1.99	23/1,274	1.28	0.66, 1.90	27/1,276	1.79	0.94, 2.64
D10	119-587	161.5	157.8, 165.2	119/1,684	6.44	4.76, 8.11*	162/1,683	9.34	7.53, 11.15	132/1,681	7.75	6.00, 9.50*	174/1,691	9.92	7.91, 11.94*	114/1,692	5.80	4.53, 7.06*
**Apolipoprotein (B) (mg/dL)**																		
All	24.0-345.0	102.7	93.7, 96.3	211/6,883	2.32	1.88, 2.76	289/6,877	3.46	2.98, 3.93	195/6,877	2.30	1.83, 2.77	324/6,895	3.73	3.18, 4.28	260/6,899	2.83	2.30, 3.36
D1	24.0-63.9	80.4	54.4, 55.8	35/613	4.18	2.27, 6.10	49/611	6.45	4.66, 8.64	24/613	3.33	1.57, 5.09	41/614	6.05	4.02, 8.08	38/616	3.78	2.34, 5.22
D10	127-345	161.5	142.6, 145.6	23/731	2.55	1.15, 3.96	24/733	2.53	1.46, 3.60	11/730	1.14	0.51, 1.78	25/732	2.87	1.52, 4.22	27/733	3.07	1.34, 4.79
**Total cholesterol (mg/dL)**																		
All	72.0-727	200.0	199.2, 200.8	909/28,688	2.28	2.06, 2.50	1,224/28,648	3.43	3.15, 3.72	916/28,686	2.60	2.31, 2.89	1,292/28,740	3.44	3.15, 3.73	1,046/28,753	2.67	2.40, 2.93
D1	72.0-150	134.7	134.1, 135.2	206/2,867	4.99	3.96, 6.02	280/2,855	8.44	7.13, 9.76	169/2,868	4.96	3.84, 6.08	244/2,872	6.92	5.79, 8.05	164/2,875	3.87	3.10, 4.63
D10	253-727	280.8	279.2, 282.5	86/3,046	2.12	1.50, 2.73†	106/3,041	3.25	2.53, 3.97†	97/3,044	2.76	2.04, 3.49†	125/3,047	3.34	2.57, 4.12†	119/3,051	3.34	2.49, 4.18
**HDL cholesterol (mg/dL)**																		
All	7.0-188	52.7	52.4, 53.1	909/28,686	2.28	2.06, 2.50	1,223/28,646	3.43	3.14, 3.71	916/28,684	2.60	2.31, 2.89	1,291/28,738	3.44	3.15, 3.73	1,045/28,751	2.66	2.39, 2.93
D1	7.0-34.3	30.4	30.2, 30.6	152/2,654	3.95	3.19, 4.71	187/2,652	5.08	4.27, 5.88	122/2,648	3.39	2.65, 4.14	193/2,662	5.32	4.39, 6.24	135/2,659	3.66	2.94, 4.39
D10	73.7-188	85.5	85.0, 86.0	74/3,130	1.93	1.38, 2.47	71/3,126	1.77	1.31, 2.24†	72/3,134	2.10	1.51, 2.69	103/3,133	2.65	1.95, 3.35†	86/3,135	2.03	1.46, 2.61†
**LDL cholesterol (mg/dL)**																		
All	21.0-344	118.2	117.3, 119.1	364/12,474	2.18	1.86, 2.50	508/12,459	3.35	2.97, 3.73	383/12,465	2.53	2.15, 2.90	556/12,503	3.51	3.10, 3.91	437/12,506	2.62	2.24, 3.00
D1	21.0-75.0	63.4	62.7, 64.0	90/1,308	5.02	3.76, 6.28	132/1,304	9.25	7.11, 11.38	88/1,309	5.82	4.06, 7.58	119/1,312	7.94	6.26, 9.62	79/1,314	4.13	3.09, 5.16
D10	163-344	185.9	184.2, 187.7	33/1,281	1.72	1.05, 2.39†	37/1,281	1.99	1.29, 2.70†	26/1,276	1.39	0.72, 2.07†	47/1,282	2.46	1.64, 3.27†	46/1,282	3.00	1.78, 4.22
**Triglycerides (mg/dL)**																		
All	14.0-3,780	140.7	137.6, 143.9	374/12,788	2.19	1.88, 2.50	524/12,774	3.39	3.02, 3.76	395/12,779	2.57	2.19, 2.95	570/12,817	3.48	3.09, 3.88	449/12,821	2.65	2.28, 3.02
D1	14.0-58.3	47.9	47.3, 48.6	18/1,161	1.08	0.42, 1.74	25/1,161	1.61	0.92, 2.30	18/1,160	1.08	0.58, 1.58	29/1,161	1.90	1.19, 2.61	21/1,161	1.41	0.80, 2.02
D10	237-3,780	382.2	364.8, 399.7	52/1,397	3.26	2.18, 4.33*	76/1,399	5.10	3.63, 6.56*	62/1,397	4.60	3.19, 6.01*	86/1,399	5.74	4.26, 7.22*	57/1,398	3.51	2.49, 4.54*

^a^Survey-weighted.

*D10 prevalence significantly higher than D1 prevalence at α = 0.05.

†D10 prevalence significantly lower than D1 prevalence at α = 0.05.

### Joint association models

CRP, glycohemoglobin, and triglycerides were considered for the final models. Although we found negative associations of TC and LDL-C with CVD, it is widely reported that the opposite relationship exists, and therefore we did not include any of the cholesterol variables in our final models [32]. Additionally, we found that participants with self-reported high cholesterol had a much higher prevalence of MI, further indicating that the negative associations observed are an artifact of the study design and not indicative of the true associations (Additional file 1: Table S1a and b and accompanying note). We eliminated fasting glucose from consideration because it was highly correlated with glycohemoglobin (r = 0.83, Additional file 1: Table S2). Furthermore, the associations between glycohemoglobin and CVD observed in the decile analysis were more robust than those involving fasting glucose (Additional file 1: Table S3). As mentioned previously, despite the robust nature of joint association models, the individual measures of association that comprise the joint estimate can still be impacted by correlation. This resulted in a three-biomarker base model for each outcome, from which backwards elimination yielded outcome-specific reduced models.

Single-biomarker models of CRP, triglycerides, and glycohemoglobin were strongly associated with each CVD, though the associations between triglycerides and stroke or MI were not significant (Table 5). The base models’ joint association estimates combining all three biomarkers (Table 6) were stronger than any estimate from the single-biomarker models. Joint associations from the base models ranged from 25.1% increased odds for CHD (OR = 1.25; 95% CI: 0.92, 1.71) to 152.5% for CHF (OR = 2.53; 95% CI: 1.86, 3.44). These estimates are smaller than the exponentiated sum of the three estimates from the single-biomarker models, consistent with the work of Winquist et al., because the single-biomarker models do not control for covariate confounding [27]. Controlling for other co-predictors in the joint association models resulted in lower individual OR estimates than in the single-biomarker models. While all but two individual ORs were significant in the single-biomarker models, seven biomarker ORs were not significant in the joint association models. The largest decrements in individual estimates occurred for CRP, with ORs decreasing an average of 16.4% from the single-biomarker models to the joint association models, compared to 6.6% for triglycerides and 1.4% for glycohemoglobin. While CRP was significantly associated with each of the CVD outcomes in the single-biomarker models, it was only significantly associated with CHF (OR = 1.86; 95% CI: 1.44, 2.43) and stroke (OR = 1.36; 95% CI: 1.07, 1.72) after controlling for triglycerides and glycohemoglobin in the joint association models. Triglycerides were strongly associated with angina in the joint association model, with an IQR increase in log-triglycerides being associated with a 23.6% increase in odds of angina (OR = 1.24 CI: 1.02, 1.50). Glycohemoglobin was significantly associated with every CVD outcome, with the odds of disease increasing between 8.5% and 16.9% for every IQR increase in log-glycohemoglobin, depending on the specific disease.

**Table 5:** **Adjusted** ^**a**^ **log-transformed single-marker logistic regression model results**

	**Cases/n**	**β x IQR**	**OR** ^**b**^	**(95% CI)**	**p-value**
**CHF**					
**Triglycerides**	352/12,561	0.2614	1.299	1.057, 1.596	0.0128
**CRP**	859/28,214	0.6057	1.833	1.538, 2.183	<0.0001
**Glycohemoglobin**	869/28,390	0.1606	1.174	1.120, 1.232	<0.0001
**Angina**					
**Triglycerides**	378/12,550	0.2520	1.287	1.067, 1.551	0.0083
**CRP**	888/28,211	0.2427	1.275	1.077, 1.509	0.0048
**Glycohemoglobin**	889/28,387	0.1430	1.154	1.099, 1.211	<0.0001
**MI**					
**Triglycerides**	550/12,587	0.1389	1.149	0.982, 1.345	0.0833
**CRP**	1,239/28,261	0.2151	1.240	1.086, 1.417	0.0015
**Glycohemoglobin**	1,256/28,436	0.1446	1.156	1.112, 1.201	<0.0001
**Stroke**					
**Triglycerides**	420/12,590	0.1297	1.139	0.952, 1.362	0.1565
**CRP**	966/28,270	0.3765	1.457	1.238, 1.715	<0.0001
**Glycohemoglobin**	985/28,447	0.1250	1.133	1.085, 1.184	<0.0001
**CHD**					
**Triglycerides**	508/12,548	0.1681	1.183	1.021, 1.371	0.0251
**CRP**	1,186/28,175	0.1953	1.216	1.037, 1.425	0.0161
**Glycohemoglobin**	1,198/28,348	0.1708	1.186	1.142, 1.232	<0.0001

^a^Models adjusted for age, sex, race/ethnicity, BMI, smoking status, systolic blood pressure, and family income.

^b^OR estimates calculated for an IQR change in the respective biomarker, or an IQR in each biomarker for the joint effect estimates. IQRs for ln(Triglycerides, mg/dL) = 0.798 [corresponding to a 2.22 fold increase in non-transformed Triglycerides], ln(CRP, mg/dL) = 2.22 [corresponding to a 9.21 fold increase in non-transformed CRP], ln(Glycohemoglobin, %) = 0.0935 [corresponding to a 1.10 fold increase in non-transformed glycohemoglobin].

**Table 6:** **Adjusted** ^**a**^ **log-transformed joint association logistic regression base model results**

**CVD outcome (cases/** ***n*** **)**	**β x IQR**	**OR** ^**b**^	**(95% CI)**	**p-value**
**CHF (351/12,531)**				
**Triglycerides**	0.1654	1.180	0.961, 1.459	0.1141
**CRP**	0.6209	1.861	1.435, 2.432	<0.0001
**Glycohemoglobin**	0.1399	1.150	1.062, 1.246	0.0006
***Joint association***	0.9262	2.525	1.856, 3.437	<0.0001
**Angina (377/12,520)**				
**Triglycerides**	0.2119	1.236	1.016, 1.504	0.0342
**CRP**	-0.0082	0.992	0.742, 1.326	0.9560
**Glycohemoglobin**	0.0974	1.102	1.021, 1.190	0.0123
***Joint association***	0.3012	1.352	1.006, 1.816	0.0456
**MI (549/12,557)**				
**Triglycerides**	0.0834	1.087	0.923, 1.280	0.3164
**CRP**	0.0478	1.049	0.822, 1.339	0.7005
**Glycohemoglobin**	0.1304	1.139	1.058, 1.227	0.0006
***Joint association***	0.2617	1.299	0.997, 1.693	0.0527
**Stroke (418/12,560)**				
**Triglycerides**	0.0686	1.071	0.888, 1.292	0.4743
**CRP**	0.3042	1.356	1.072, 1.715	0.0112
**Glycohemoglobin**	0.0812	1.085	1.010, 1.164	0.0248
***Joint association***	0.4538	1.575	1.194, 2.077	0.0013
**CHD (508/12,518)**				
**Triglycerides**	0.1082	1.114	0.965, 1.287	0.1419
**CRP**	-0.0404	0.960	0.719, 1.284	0.7851
**Glycohemoglobin**	0.1560	1.169	1.092, 1.251	<0.0001
***Joint association***	0.2238	1.251	0.918, 1.705	0.1565

^a^Models adjusted for age, gender, race/ethnicity, BMI, smoking status, and family income.

^b^OR estimates calculated for an IQR change in the respective biomarker, or an IQR in each biomarker for the joint effect estimates. IQRs for ln(Triglycerides, mg/dL) = 0.798 [corresponding to a 2.22 fold increase in non-transformed Triglycerides], ln(CRP, mg/dL) = 2.22 [corresponding to a 9.21 fold increase in non-transformed CRP], ln(Glycohemoglobin, %) = 0.0935 [corresponding to a 1.10 fold increase in non-transformed glycohemoglobin].

After removing nonsignificant biomarkers from each model, we arrived at a unique reduced model for each outcome. CHF and stroke joint association models included CRP and glycohemoglobin; the angina joint association model included triglycerides and glycohemoglobin; and MI and CHD models included only glycohemoglobin (Table 7). The ORs for the fully adjusted reduced joint association models were all significant, indicating that a joint increase in serum concentrations of the selected log-transformed biomarkers was associated with an increase in the odds of the corresponding cardiovascular outcome. Specifically, joint IQR increases in log-CRP and log-glycohemoglobin were associated with increased odds of CHF (OR = 2.03; 95% CI: 1.70, 2.42) and stroke (OR = 1.58; 95% CI: 1.34-1.87), and a joint increase in log-triglycerides and log-glycohemoglobin was associated with increased odds of angina (OR = 1.36; 95% CI: 1.13, 1.65). Log-transformed glycohemoglobin was the only biomarker that was significant in every outcome-specific reduced model. Neither triglycerides nor CRP were significant for MI or CHD, such that the reduced models were single-biomarker glycohemoglobin models. For those single-biomarker models, the OR for an IQR increase in log-glycohemoglobin was 1.16 (95% CI: 1.11, 1.20) for MI and 1.19 (95% CI: 1.14, 1.23) for CHD.

**Table 7:** **Adjusted** ^**a**^ **log-transformed reduced joint association logistic regression model results**

	**CVD outcome (cases/** ***n*** **)**	**β x IQR**	**OR** ^**b**^	**(95% CI)**	**p-value**
**CHF** (858/28,163)					
**CRP**		0.5647	1.759	1.473, 2.100	<0.0001
**Glycohemoglobin**		0.1412	1.152	1.096, 1.210	<0.0001
**Joint association**		0.7059	2.026	1.699, 2.415	<0.0001
**Angina** (378/12,523)					
**Triglycerides**		0.2133	1.235	1.018, 1.499	0.0323
**Glycohemoglobin**		0.0972	1.102	1.022, 1.188	0.0114
**Joint association**		0.3086	1.362	1.126, 1.646	0.0015
**MI** (1,256/28,436)					
**Glycohemoglobin**		0.1446	1.156	1.112, 1.201	<0.0001
**Stroke** (963/28,219)					
**CRP**		0.3429	1.409	1.197, 1.659	<0.0001
**Glycohemoglobin**		0.1165	1.124	1.076, 1.173	<0.0001
**Joint association**		0.4594	1.583	1.338, 1.873	<0.0001
**CHD** (1,198/28,348)					
**Glycohemoglobin**		0.1708	1.186	1.142, 1.232	<0.0001

^a^Models adjusted for age, sex, race/ethnicity, BMI, smoking status, systolic blood pressure, and family income.

^b^OR estimates calculated for an IQR change in the respective biomarker, or an IQR in each biomarker for the joint association estimates. IQRs for ln(Triglycerides, mg/dL) = 0.798 [corresponding to a 2.22 fold increase in non-transformed Triglycerides], ln(CRP, mg/dL) =2.22 [corresponding to a 9.21 fold increase in non-transformed CRP], ln(Glycohemoglobin, %)=0.0935 [corresponding to a 1.10 fold increase in non-transformed glycohemoglobin].

In addition to the base and reduced models, we used age-stratified models to examine age-related differences in biomarker-CVD associations. The lowest age groups (20–34 and 35–44) had such a limited number of cardiovascular events that most estimates were imprecise and unreliable (Additional file 1: Tables S3a and S4e). The associations in the older age groups (45–60 and 60+) did not deviate noticeably from the nonstratified models.

## Discussion

Our study explored associations between serum biomarkers and CVD. Using a joint association approach to logistic regression modeling, we saw a variety of associations of individual and joint biomarkers with CVD. Consistent with previous studies, we found that single-biomarker models of CRP, triglycerides, and glycohemoglobin were significantly associated with CVD [33-37]. However, we found that the magnitude and significance of these associations decreased after controlling for covariate confounding of the selected biomarkers in the joint association models. This demonstrates the likely presence of confounding among the biomarkers; the potential exists for overestimation of the association between individual biomarkers and CVD when failing to adjust for other co-varying biomarkers.

Biomarker associations with CVD were overestimated when using single-biomarker models in comparison with the full models. While single-marker CRP models showed strong associations with angina, MI, and CHD, those results were no longer significant when controlling for triglycerides and glycohemoglobin. While a number of studies have shown a strong association between CRP and CVD, there is evidence that the relationship may lessen in relation to diabetes status [33]. A 2002 long-term follow-up case control study by Sakkinen et al. found that the predictive effect of CRP for MI was diminished in men with diabetes [35]. Sakkinen et al. hypothesized that this attenuation was likely due to an overlap in information between CRP concentrations and diabetes diagnosis, which is supported by multiple studies that found significant correlations among CRP, diabetes, and other features of MetS [34,38,39]. These concerns have also been raised regarding evidence supporting an association between triglycerides and CHD. In a review of the literature, Sarwar et al. concluded that associations between triglycerides and CHD remain uncertain due to potential codependence of other risk factors, such as other lipids [40]. Our findings substantiated this uncertainty, as a significant association between triglycerides and CHD in the single-biomarker model was no longer significant when controlling for CRP and glycohemoglobin. These examples demonstrate the advantage of a multivariate approach. While these biomarkers on their own can be important predictors of CVD, by controlling for confounding between the biomarkers, it may be possible to achieve a more accurate evaluation of how biomarkers affect CVD risk on an individual basis.

Glycohemoglobin was the only biomarker in our analyses that was significantly associated with MI or CHD, and thus it was the only biomarker to be significantly associated with every CVD outcome in the reduced joint association models. This is consistent with existing evidence of an association between diabetes mellitus and CVD. A review of epidemiologic studies shows both cross-sectional associations and prospective temporal relationships between diabetes and CVD incidence and mortality [41]. Our study also found that of all variations of CVD examined, glycohemoglobin had the strongest association with CHD, which has previously been established as the most common CVD outcome in adults with diabetes [41,42].

Although the individual biomarkers are associated with the CVD tested here, the reduced joint association modeling results suggest that unique combinations of biomarkers with their related measures of association for each model can be used to produce a unique risk estimate for each CVD. For example, CRP and glycohemoglobin were jointly associated with CHF and stroke, whereas triglycerides and glycohemoglobin were jointly associated with angina. Hence, where biomarkers have served as general indicators of CVD risk, joint models can be utilized to indicate risk for specific CVDs. Moreover, the reduced joint association models indicated large increases in CVD odds for joint increases in biomarker concentrations that were larger than the OR estimate from any single marker within that model but still lower than if the overall association were estimated from single-biomarker models. As in the individual biomarker estimates, controlling for co-predictor confounding prevents an overestimation of the joint biomarker association [27].

Our conclusions are limited by several factors of our study design. As with all cross-sectional studies, we are unable to examine temporality between the biomarkers and outcomes. Thus, incidence of disease cannot be assessed here. Using a proxy measure creates the potential for subjects with recently increased or decreased biomarker concentrations to have measured levels that do not match their historical exposure. This could be particularly relevant to patients who have reported CVD but are currently taking medications or undergoing other health interventions, including improved diet and exercise, that may have lowered their biomarker levels. This potential differential exposure misclassification may have led to observed odds ratios that underestimate the true magnitude of the associations [43]. Observed ORs may also be underestimated due to survival bias, whereby high biomarker levels may be predictive of CVD mortality [44-47]. We speculate that the negative associations seen between CVD prevalence and TC and LDL-C levels are at least partially the result of survival bias and/or the use cholesterol medication post-CVD diagnosis. Another potential limitation of the lack of temporality is the possibility that CRP levels were elevated by post-event inflammation in participants who reported CVD [48], resulting in CRP associations biased away from the null.

The age-stratified analysis was hindered by a low number of CVD cases in the younger age groups, resulting in imprecise and unreliable ORs, making it difficult to infer potential relationships among age, biomarker levels, and CVD. Sex-related differences were not explored for this project, because division by sex would have further reduced the numbers of cases. Additionally, CVD status was self-reported and thus was not verified. In general, self-reporting of disease status and exposures, including medication, diet, and exercise, add uncertainty to the analysis. Although we do not expect the accuracy of self-reporting to vary across biomarker levels, it is possible that self-reporting accuracy varied on some factor simultaneously affecting biomarker levels, which may have introduced unknown bias into our measures of association. However, the analysis presented here does not presume to judge causality between self-reported disease status and the combinations of biomarkers tested here. This analysis reports associations for the purpose of estimating risk.

Despite these limitations, our study had a number of strengths. To our knowledge, this is the first large-scale analysis using a joint association approach to assessing the relationship between multiple biomarker concentrations and CVD. NHANES provides a nationally representative sample of the US, such that our results can be generalized to the US adult civilian population. The large sample size of NHANES lends confidence to the assessment that helps to offset the uncertainties listed above. Given the consistency of NHANES sample design and data collection methodology, our results can provide a basis for comparison when analyzing relationships between CVD and biomarkers among future cohorts.

## Conclusions

Our work has built upon evidence from a multitude of previous studies that have demonstrated associations between triglycerides, CRP, and glycohemglobin and variations of CVD. Specifically, this study highlights the need to consider a joint effects approach to determining both individual biomarker associations as well as the impact of simultaneous increases in multiple biomarker concentrations. This approach may lead to more accurate individual biomarker risk estimation through co-predictor confounding control, a cumulative perspective of the impact of related biomarkers on CVD, and the potential to observe unique combinations of biomarkers that may be predictive of variations of CVD. Future longitudinal studies on the joint effect of multiple biomarkers on CVD are needed to assess temporal relationships and determine whether these models can be developed to predict future onset of CVD. Additionally, expanding joint association models to include interaction terms could provide detail as to how the joint associations vary given specific combinations of biomarkers (i.e., high CRP vs. low glycohemoglobin).

## Additional file

Additional file 1:
**Multiple biomarker models for improved risk estimation of specific cardiovascular diseases related to metabolic syndrome: a cross-sectional study.**

## Abbreviations

ApoB: Apolipoprotein B
ATP III: Adult Treatment Panel III
CI: Confidence interval
CHD: Coronary heart disease
CHF: Congestive heart failure
CRP: C-reactive protein
CVD: Cardiovascular disease
HDL-C: High-density lipoprotein cholesterol
IQR: Interquartile range
LDL-C: Low-density lipoprotein cholesterol
MetS: Metabolic syndrome
MI: Myocardial infarction
NHANES: National Health and Nutrition Examination Survey
OR: Odds ratio
SBP: Systolic blood pressure
TC: Total cholesterol
