# Supplementary material for: Multiple biomarker models for improved risk estimation of specific cardiovascular diseases related to metabolic syndrome: a cross-sectional study
Source: Popul Health Metr. 2015 Mar 14;13:7. doi: 10.1186/s12963-015-0041-5 (PMC4363346; doi:10.1186/s12963-015-0041-5)
Supplement: Additional file 1: — Multiple biomarker models for improved risk estimation of specific cardiovascular diseases related to metabolic syndrome: a cross-sectional study. [file 12963_2015_41_MOESM1_ESM.docx]

**Supplemental Material**

**Multiple biomarker models for improved risk estimation of specific cardiovascular diseases related to metabolic syndrome: a cross-sectional study**

**Model Description**

The following equations (1-5 and accompanying descriptions) for estimating the joint association of biomarkers, including odds ratios (ORs) and 95% confidence intervals (CIs), were adapted from Winquist et al. (2013). The models for estimating the joint association of *b* log-transformed biomarkers with the health outcomes of interest (Y) were as follows:

$$\left( 1 \right) Log\left[ E\left( Y \right) \right]= \propto+ \sum_{i=1}^{b} \beta_{i}(biomarker)_{i}+\sum_{j} \gamma_{j}(sex)_{j}+\sum_{k} {\delta\gamma}_{k}(age)_{k}+ \sum_{l} \varepsilon_{l}(race/ethncity)_{l}+\sum_{m} {\vartheta\gamma}_{m}(BMI)_{m}+\sum_{n} \tau_{n}(smoking history)_{n}+\sum_{p} \varphi_{p}(SBP)_{p}+\sum_{q} \omega_{q}(income)_{q}$$

where the dependent variable (Y) is the health outcome of interest (e.g., congestive heart failure, angina, heart attack, stroke, or coronary heart disease). The model included indicator variables for sex, age, race/ethnicity, BMI, smoking history, systolic blood pressure (SBP), and family income.

Joint association ORs, measuring the odds of disease for a simultaneous interquartile range (IQR) increase in each log-transformed biomarker, were calculated by taking the exponentiated sum of the product of each log-transformed biomarker regression coefficient and that respective log-transformed biomarker’s IQR. This was done using the following formulas, as corresponds to the model in equation 1:

$$\left( 2 \right) L^{'}\beta=\sum_{i=1}^{b} \beta_{i}{(biomarker IQR)}_{i}$$

$$\left( 3 \right) OR_{JA}=exp(L'\beta)$$

where the joint association of *b* log-transformed biomarkers is estimated as OR_JA_. $\beta_{i}$ is the estimated coefficient for a given log-transformed biomarker (*b*), $\beta$ is the vector of the combined estimates, and L is the vector of the respective log-transformed biomarker IQRs.

The 95% CI corresponding to the OR estimate in equation 3 was calculated using the SAS v 9.3 (SAS Institute Inc., Cary, N.C.) “proc genmod” “estimate” statement, which is generated using the following equations:

(4) $se_{L'\beta}= \sqrt{L'\hat{\sum}L}$

$$\left( 5 \right) 95\% CI_{{OR}_{JA}}=(\exp\left( L^{'}\beta-Z_{1-\frac{\alpha}{2}}se_{L^{'}\beta} \right),\exp\left( L^{'}\beta+Z_{1-\frac{\alpha}{2}}se_{L^{'}\beta} \right))$$

where $\hat{\sum}$ is the estimated covariance matrix of the paramter estimates and α=0.05.

| **Supplemental Table 1a. MI Prevalence by self-reported high cholesterol status and mean total cholesterol levels** | | | | |
| --- | --- | --- | --- | --- |
| **Doctor Diagnosed High Cholesterol** | **Prevalent MI** | **N** | **Within Group MI Prevalence (%)*** | **Mean TC^1^** |
| Yes | Yes | 740 | 7.18 | 192.7 |
|  | No | 7,528 | - | 216.5 |
| No | Yes | 412 | 2.68 | 181.4 |
|  | No | 10,954 | - | 195.5 |
| ^1^Survey-weighted |  |  |  |  |
|  |  |  |  |  |
|  |  |  |  |  |
| **Supplemental Table 1b. MI Prevalence by self-reported high cholesterol status and mean LDL cholesterol levels** | | | | |
| **Doctor Diagnosed High Cholesterol** | **Prevalent MI** | **N^1^** | **Within Group MI Prevalence (%)*** | **Mean LDL^2^** |
| Yes | Yes | 312 | 7.32 | 104.3 |
|  | No | 3,628 | - | 130.1 |
| No | Yes | 190 | 2.69 | 114.4 |
|  | No | 4,826 | - | 106.8 |
| ^1^Population subsample |  |  |  |  |
| ^2^Survey-weighted |  |  |  |  |

Note: Supplementary Tables 1a and 1b show that participants who answer “Yes” to the question “have you ever been told by a doctor or healthcare professional that your cholesterol level was high?” have a much higher prevalence of MI than those who answer “No”. This is consistent with research that shows a positive association between cholesterol levels and CVD^[[1]](#footnote-1)^, and is what we expected to see within the examination of CVD prevalence by cholesterol deciles. The surprising relationship displayed in these tables is that participants with prevalent MI generally have lower mean cholesterol levels than those without, which is also reflected in the deciles tables. We speculate that this negative association is the result of the cross-sectional study design. Because the study design only allows for analysis of prevalence and not incidence, participants who have had a CVD event may be taking cholesterol lowering medication, and/or cholesterol levels at the time of the event may be a predictor (or surrogate predictor) of CVD mortality, resulting in survival bias.

| **Supplemental Table 2. Pearson Correlation Coefficients for Biomarkers of Interest** | | | | |
| --- | --- | --- | --- | --- |
|  | Triglyceride | C-reactive protein | Glycohemo-globin | Fasting Glucose |
| Triglyceride | 1 | -- | -- | -- |
| C-reactive protein | 0.0324 | 1 | -- | -- |
| Glycohemoglobin | 0.1996 | 0.1018 | 1 | -- |
| Fasting Glucose | 0.2299 | 0.0595 | 0.8317 | 1 |

| **Supplemental Table 3. Summary Statistics and Survey-Weighted CVD Prevalence by Survey-Weighted Deciles of Glycohemoglobin and Fasting Glucose** | | | | | | | | | | | | | | | | | | | | | | | | |  | |
| --- | --- | --- | --- | --- | --- | --- | --- | --- | --- | --- | --- | --- | --- | --- | --- | --- | --- | --- | --- | --- | --- | --- | --- | --- | --- | --- |
|  | **Summary Statistics** | | |  | **Congestive Heart Failure** | | |  | **Coronary Heart Disease** | | |  | **Angina** | | |  | **Heart Attack** | | |  | **Stroke** | | |  | |  |
|  | **Range** | **Mean^a^** | **95% CI^a^** |  | **n (case/total)** | **%^a^** | **95% CI^a^** |  | **n (case/total)** | **%^a^** | **95% CI^a^** |  | **n (case/total)** | **%^a^** | **95% CI^a^** |  | **n (case/total)** | **%^a^** | **95% CI^a^** |  | **n (case/total)** | **%^a^** | **95% CI^a^** |  | |  |
| **Glycohemoglobin** | |  |  |  |  |  |  |  |  |  |  |  |  |  |  |  |  |  |  |  |  |  |  |  | |  |
| All | 2.00-18.80 | 5.51 | 5.49, 5.53 |  | 926/29,000 | 2.30 | 2.08, 2.53 |  | 1,242/28,958 | 3.44 | 3.15, 3.72 |  | 932/28,999 | 2.63 | 2.34, 2.92 |  | 1,313/29,051 | 3.45 | 3.16, 3.75 |  | 1,074/29,066 | 2.71 | 2.44, 2.98 |  | |  |
| D1 | 2.00-4.83 | 4.67 | 4.66, 4.68 |  | 21/2,225 | 0.89 | 0.42, 1.36 |  | 28/2,227 | 0.87 | 0.44, 1.29 |  | 16/2,227 | 0.75 | 0.34, 1.16 |  | 34/2,227 | 1.16 | 0.63, 1.68 |  | 38/2,227 | 1.44 | 0.80, 2.08 |  | |  |
| D2 | 4.84-4.98 | 4.90 | 4.90, 4.90 |  | 10/1,241 | 0.61 | 0.10, 1.12 |  | 17/1,242 | 1.17 | 0.48, 1.86 |  | 14/1,241 | 0.72 | 0.25, 1.19 |  | 16/1,240 | 0.82 | 0.29, 1.35 |  | 15/1,242 | 0.90 | 0.27, 1.54 |  | |  |
| D3 | 4.99-5.09 | 5.00 | 5.00, 5.00 |  | 18/1,678 | 0.89 | 0.45, 1.34 |  | 24/1,678 | 1.04 | 0.60, 1.47 |  | 13/1,682 | 0.65 | 0.23, 1.07 |  | 15/1,682 | 0.77 | 0.40, 1.13 |  | 20/1,683 | 0.79 | 0.42, 1.16 |  | |  |
| D4 | 5.10-5.20 | 5.10 | 5.10, 5.10 |  | 29/2,145 | 1.00 | 0.60, 1.40 |  | 35/2,144 | 1.03 | 0.55, 1.51 |  | 30/2,145 | 0.92 | 0.52, 1.32 |  | 45/2,147 | 1.35 | 0.81, 1.88 |  | 39/2,146 | 1.30 | 0.87, 1.73 |  | |  |
| D5 | 5.21-5.30 | 5.20 | 5.20, 5.20 |  | 45/2,607 | 1.13 | 0.77, 1.49 |  | 73/2,606 | 2.49 | 1.79, 3.19 | * | 53/2,607 | 1.83 | 1.21, 2.45 | * | 69/2,607 | 2.23 | 1.66, 2.81 |  | 71/2,604 | 2.18 | 1.60, 2.76 |  | |  |
| D6 | 5.31-5.40 | 5.30 | 5.30, 5.30 |  | 44/2,729 | 1.25 | 0.86, 1.63 |  | 85/2,725 | 2.38 | 1.76, 3.00 | * | 62/2,730 | 1.78 | 1.30, 2.25 | * | 82/2,732 | 2.30 | 1.66, 2.95 |  | 73/2,733 | 1.79 | 1.25, 2.33 |  | |  |
| D7 | 5.41-5.49 | 5.40 | 5.40, 5.40 |  | 67/2,702 | 2.05 | 1.46, 2.64 | * | 89/2,692 | 2.94 | 2.07, 3.81 | * | 64/2,698 | 2.46 | 1.77, 3.15 | * | 101/2,699 | 3.16 | 2.39, 3.92 | * | 77/2,703 | 2.13 | 1.56, 2.71 |  | |  |
| D8 | 5.50-5.68 | 5.54 | 5.54, 5.55 |  | 130/4,562 | 2.08 | 1.60, 2.55 | * | 199/4,549 | 3.90 | 3.20, 4.60 | * | 144/4,562 | 2.92 | 2.21, 3.63 | * | 197/4,574 | 3.73 | 3.15, 4.31 | * | 141/4,574 | 2.40 | 1.96, 2.84 |  | |  |
| D9 | 5.69-6.06 | 5.81 | 5.80, 5.81 |  | 199/4,721 | 3.47 | 2.94, 4.00 | * | 277/4,716 | 5.17 | 4.38, 5.97 | * | 221/4,721 | 4.19 | 3.53, 4.84 | * | 298/4,734 | 5.42 | 4.62, 6.22 | * | 258/4,737 | 4.60 | 3.91, 5.29 | * | |  |
| D10 | 6.07-18.80 | 7.34 | 7.30, 7.42 |  | 363/4,390 | 7.47 | 6.44, 8.49 | * | 415/4,379 | 9.69 | 8.48, 10.91 | * | 315/4,386 | 7.13 | 5.94, 8.32 | * | 456/4,409 | 9.70 | 8.60, 10.80 | * | 342/4,417 | 6.98 | 6.04, 7.93 | * | |  |
| **Fasting Glucose** | |  |  |  |  |  |  |  |  |  |  |  |  |  |  |  |  |  |  |  |  |  |  |  | |  |
| All | 38.0-587.3 | 102.7 | 102.0, 103.4 |  | 377/12,929 | 2.18 | 1.87, 2.48 |  | 528/12,915 | 3.38 | 3.01, 3.74 |  | 401/12.920 | 2.60 | 2.21, 2.98 |  | 578/12,957 | 3.48 | 3.08, 3.88 |  | 457/12,962 | 2.70 | 2.33, 3.07 |  | |  |
| D1 | 38.00-84.9 | 80.4 | 80.0, 80.7 |  | 23/1,272 | 1.30 | 0.70, 1.91 |  | 23/1,269 | 1.70 | 0.77, 2.62 |  | 17/1,271 | 1.24 | 0.49, 1.99 |  | 23/1,274 | 1.28 | 0.66, 1.90 |  | 27/1,276 | 1.79 | 0.94, 2.64 |  | |  |
| D2 | 85.0-88.9 | 86.9 | 86.8, 87.0 |  | 12/1,126 | 0.84 | 0.11, 1.57 |  | 15/1,126 | 0.90 | 0.34, 1.45 |  | 13/1,124 | 1.14 | 0.42, 1.86 |  | 12/1,128 | 0.84 | 0.28, 1.39 |  | 25/1,128 | 1.68 | 0.80, 2.57 |  | |  |
| D3 | 89.0-91.9 | 90.2 | 90.2, 90.3 |  | 22/1,195 | 1.29 | 0.59, 2.00 |  | 16/1,195 | 1.04 | 0.44, 1.63 |  | 14/1,197 | 0.87 | 0.35, 1.40 |  | 26/1,197 | 1.48 | 0.87, 2.09 |  | 22/1,198 | 1.38 | 0.64, 2.11 |  | |  |
| D4 | 92.0-94.7 | 93.1 | 93.1, 93.2 |  | 15/1,233 | 1.20 | 0.51, 1.89 |  | 36/1,234 | 2.03 | 1.27, 2.79 |  | 26/1,234 | 1.85 | 0.89, 2.81 |  | 35/1,235 | 1.84 | 1.20, 2.49 |  | 28/1,236 | 1.85 | 1.06, 2.64 |  | |  |
| D5 | 94.8-97.0 | 95.7 | 95.6, 95.7 |  | 26/1,012 | 1.80 | 0.94, 2.65 |  | 33/1,010 | 2.64 | 1.54, 3.73 |  | 24/1,012 | 1.70 | 0.85, 2.56 |  | 34/1,014 | 2.15 | 1.30, 3.00 |  | 25/1,014 | 1.78 | 0.92, 2.64 |  | |  |
| D6 | 97.1-99.9 | 98.2 | 98.1, 98.3 |  | 23/1,298 | 1.08 | 0.50, 1.66 |  | 45/1,297 | 2.98 | 1.83, 4.14 |  | 34/1,296 | 2.15 | 1.21, 3.09 |  | 55/1,298 | 3.44 | 2.11, 4.76 | * | 42/1,298 | 2.53 | 1.62, 3.45 |  | |  |
| D7 | 100.0-103.1 | 101.4 | 101.4, 101.5 |  | 38/1,362 | 2.25 | 1.38, 3.11 |  | 50/1,356 | 3.08 | 2.12, 4.05 |  | 43/1,362 | 2.82 | 1.88, 3.77 |  | 60/1,363 | 3.93 | 2.64, 5.22 | * | 45/1,363 | 2.57 | 1.73, 3.40 |  | |  |
| D8 | 103.2-108.0 | 105.4 | 105.3, 105.4 |  | 28/1,221 | 1.79 | 1.07, 2.51 |  | 51/1,218 | 3.83 | 2.42, 5.24 |  | 31/1,219 | 2.29 | 1.39, 3.20 |  | 48/1,224 | 3.39 | 2.06, 4.72 | * | 46/1,223 | 3.24 | 2.00, 4.48 |  | |  |
| D9 | 108.1-119.3 | 112.4 | 112.2, 112.6 |  | 71/1,526 | 3.69 | 2.61, 4.78 | * | 97/1,527 | 6.06 | 4.75, 7.36 | * | 67/1,524 | 3.91 | 2.90, 4.92 | * | 111/1,533 | 6.12 | 4.76, 7.49 | * | 83/1,534 | 4.22 | 3.12, 5.31 | * | |  |
| D10 | 119.4-587.3 | 161.5 | 157.8, 165.2 |  | 119/1,684 | 6.44 | 4.76, 8.11 | * | 162/1,683 | 9.34 | 7.53, 11.15 | * | 132/1,681 | 7.75 | 6.00, 9.50 | * | 174/1,691 | 9.92 | 7.91, 11.94 | * | 114/1,692 | 5.80 | 4.53, 7.06 | * | |  |
| ^a^Survey-Weighted | |  |  |  |  |  |  |  |  |  |  |  |  |  |  |  |  |  |  |  |  |  |  |  | |  |
| *D10 Prevalence Significantly Higher than D1 Prevalence at α=0.05 | | | | | | | | | |  |  |  |  |  |  |  |  |  |  |  |  |  |  |  | |  |

| **Supplemental Table 4a. Age-Stratified Adjusted^a^ Log-Transformed Joint Association Logistic Regression Model Results - Congestive Heart Failure** | | | | | | |
| --- | --- | --- | --- | --- | --- | --- |
|  | **cases/*n*** |  | **β*IQR** | **OR^b^** | **(95% CI)** | **p-value** |
| **Congestive Heart Failure** | | | | | | |
| **Ages 20-34** | 6/3,331 |  |  |  |  |  |
| **Triglycerides** |  |  | 0.6667 | 1.948 | 0.663, 5.721 | 0.2252 |
| **CRP** |  |  | 1.4146 | 4.115 | 0.866, 19.549 | 0.0752 |
| **Glycohemoglobin** |  |  | -0.1727 | 0.841 | 0.539, 1.314 | 0.4479 |
| **Joint Association** |  |  | 1.9086 | 6.744 | 0.7236, 2.937 | 0.0940 |
| **Ages 35-44** | 16/2,121 |  |  |  |  |  |
| **Triglycerides** |  |  | 0.6079 | 1.837 | 1.162, 2.904 | 0.0093 |
| **CRP** |  |  | 0.9341 | 2.545 | 0.952, 6.806 | 0.0627 |
| **Glycohemoglobin** |  |  | -0.4754 | 0.622 | 0.320, 1.209 | 0.1612 |
| **Joint Association** |  |  | 1.0666 | 2.906 | 0.808, 10.451 | 0.1023 |
| **Ages 45-60** | 68/3,157 |  |  |  |  |  |
| **Triglycerides** |  |  | 0.2616 | 1.299 | 0.902, 1.871 | 0.1600 |
| **CRP** |  |  | 0.8746 | 2.398 | 1.208, 4.759 | 0.0124 |
| **Glycohemoglobin** |  |  | 0.1514 | 1.164 | 1.021, 1.326 | 0.0231 |
| **Joint Association** |  |  | 1.2876 | 3.624 | 1.719, 7.639 | 0.0007 |
| **Ages 65+** | 261/3,922 |  |  |  |  |  |
| **Triglycerides** |  |  | 0.0055 | 1.006 | 0.786, 1.287 | 0.9649 |
| **CRP** |  |  | 0.5375 | 1.712 | 1.271, 2.305 | 0.0004 |
| **Glycohemoglobin** |  |  | 0.1674 | 1.182 | 1.075, 1.300 | 0.0006 |
| **Joint Association** |  |  | 0.7104 | 2.035 | 1.437, 2.882 | <0.0001 |
| ^a^Models adjusted for age, gender, race/ethnicity, BMI, smoking status, and family income.  ^b^OR estimates calculated for an IQR change in the respective biomarker, or an IQR in each biomarker  for the joint association estimates. IQRs for ln(Triglycerides, mg/dL) = 0.798 [corresponding to a 2.22 fold increase in non-transformed Triglycerides], ln(CRP, mg/dL) = 2.22 [corresponding to a 9.21 fold increase in non-transformed CRP], ln(Glycohemoglobin, %) = 0.0935 [corresponding to a 1.10 fold increase in non-transformed glycohemoglobin]. | | | | | | |

| **Supplemental Table 4b. Age-Stratified Adjusted^a^ Log-Transformed Joint Association Logistic Regression Model Results - Angina** | | | | | | |
| --- | --- | --- | --- | --- | --- | --- |
|  | **cases/*n*** |  | **β*IQR** | **OR^b^** | **(95% CI)** | **p-value** |
| **Angina** | | | | | | |
| **Ages 20-34** | 4/3,329 |  |  |  |  |  |
| **Triglycerides** |  |  | -1.1548 | 0.315 | 0.122, 0.815 | 0.0172 |
| **CRP** |  |  | 0.4967 | 1.645 | 0.090, 30.065 | 0.7372 |
| **Glycohemoglobin** |  |  | 0.1364 | 1.146 | 0.487, 2.700 | 0.7550 |
| **Joint Association** |  |  | -0.5208 | 0.594 | 0.012, 30.077 | 0.7948 |
| **Ages 35-44** | 17/2,119 |  |  |  |  |  |
| **Triglycerides** |  |  | 0.2089 | 1.232 | 0.430, 3.531 | 0.6972 |
| **CRP** |  |  | 2.0788 | 7.995 | 3.133, 20.398 | <0.0001 |
| **Glycohemoglobin** |  |  | 0.0237 | 1.024 | 0.791, 1.326 | 0.8576 |
| **Joint Association** |  |  | 2.3114 | 10.088 | 3.278, 31.047 | <0.0001 |
| **Ages 45-60** | 83/3,153 |  |  |  |  |  |
| **Triglycerides** |  |  | 0.1439 | 1.273 | 0.960, 1.688 | 0.0935 |
| **CRP** |  |  | 0.2859 | 0.904 | 0.516, 1.584 | 0.7248 |
| **Glycohemoglobin** |  |  | 0.0726 | 1.007 | 1.007, 1.338 | 0.0403 |
| **Joint Association** |  |  | 0.2784 | 0.774 | 0.774, 2.305 | 0.2984 |
| **Ages 65+** | 273/3,919 |  |  |  |  |  |
| **Triglycerides** |  |  | 0.2133 | 1.238 | 0.970, 1.580 | 0.0864 |
| **CRP** |  |  | -0.2587 | 0.772 | 0.549, 1.087 | 0.1378 |
| **Glycohemoglobin** |  |  | 0.0557 | 1.057 | 0.971, 1.152 | 0.2018 |
| **Joint Association** |  |  | 0.0103 | 1.010 | 0.676, 1.511 | 0.9599 |
| ^a^Models adjusted for age, gender, race/ethnicity, BMI, smoking status, and family income.  ^b^OR estimates calculated for an IQR change in the respective biomarker, or an IQR in each  biomarker for the joint association estimates. IQRs for ln(Triglycerides, mg/dL) = 0.798 [corresponding to a 2.22 fold increase in non-transformed Triglycerides], ln(CRP, mg/dL) = 2.22 [corresponding to a 9.21 fold increase in non-transformed CRP], ln(Glycohemoglobin, %) = 0.0935 [corresponding to a 1.10 fold increase in non-transformed glycohemoglobin]. | | | | | | |

| **Supplemental Table 4c. Age-Stratified Adjusted^a^ Log-Transformed Joint Association Logistic Regression Model Results - Heart Attack** | | | | | | |
| --- | --- | --- | --- | --- | --- | --- |
|  | **cases/*n*** |  | **β*IQR** | **OR^b^** | **(95% CI)** | **p-value** |
| **Heart Attack** | | | | | | |
| **Ages 20-34** | 11/3,331 |  |  |  |  |  |
| **Triglycerides** |  |  | -0.2567 | 0.774 | 0.293, 2.041 | 0.6039 |
| **CRP** |  |  | 0.6138 | 1.848 | 0.981, 3.480 | 0.0575 |
| **Glycohemoglobin** |  |  | 0.3752 | 1.455 | 0.956, 2.216 | 0.0802 |
| **Joint Association** |  |  | 0.7323 | 2.080 | 0.901, 4.801 | 0.0862 |
| **Ages 35-44** | 25/2,122 |  |  |  |  |  |
| **Triglycerides** |  |  | 0.2517 | 1.286 | 0.718, 2.305 | 0.3978 |
| **CRP** |  |  | 0.9689 | 2.635 | 0.972, 7.145 | 0.0569 |
| **Glycohemoglobin** |  |  | -0.0160 | 0.984 | 0.819, 1.183 | 0.8648 |
| **Joint Association** |  |  | 1.2046 | 3.336 | 1.202, 9.258 | 0.0207 |
| **Ages 45-60** | 115/3,162 |  |  |  |  |  |
| **Triglycerides** |  |  | 0.0845 | 1.088 | 0.844, 1.403 | 0.5150 |
| **CRP** |  |  | 0.0201 | 1.020 | 0.617, 1.689 | 0.9378 |
| **Glycohemoglobin** |  |  | 0.1500 | 1.162 | 1.029, 1.312 | 0.0158 |
| **Joint Association** |  |  | 0.2546 | 1.290 | 0.783, 2.125 | 0.3174 |
| **Ages 65+** | 398/3,942 |  |  |  |  |  |
| **Triglycerides** |  |  | 0.0758 | 1.079 | 0.858, 1.356 | 0.5166 |
| **CRP** |  |  | -0.0521 | 0.949 | 0.689, 1.308 | 0.7504 |
| **Glycohemoglobin** |  |  | 0.1215 | 1.129 | 1.033, 1.235 | 0.0077 |
| **Joint Association** |  |  | 0.1452 | 1.156 | 0.809, 1.653 | 0.4255 |
| ^a^Models adjusted for age, gender, race/ethnicity, BMI, smoking status, and family income.  ^b^OR estimates calculated for an IQR change in the respective biomarker, or an IQR in each  biomarker for the joint association estimates. IQRs for ln(Triglycerides, mg/dL) = 0.798 [corresponding to a 2.22 fold increase in non-transformed Triglycerides], ln(CRP, mg/dL) = 2.22 [corresponding to a 9.21 fold increase in non-transformed CRP], ln(Glycohemoglobin, %) = 0.0935 [corresponding to a 1.10 fold increase in non-transformed glycohemoglobin]. | | | | | | |

| **Supplemental Table 4d. Age-Stratified Adjusted^a^ Log-Transformed Joint Association Logistic Regression Model Results - Stroke** | | | | | | |
| --- | --- | --- | --- | --- | --- | --- |
|  | **cases/*n*** |  | **β*IQR** | **OR^b^** | **(95% CI)** | **p-value** |
| **Stroke** | | | | | | |
| **Ages 20-34** | 9/3,331 |  |  |  |  |  |
| **Triglycerides** |  |  | -0.8287 | 0.437 | 0.087, 2.189 | 0.3137 |
| **CRP** |  |  | 0.5696 | 1.768 | 0.780, 4.007 | 0.1724 |
| **Glycohemoglobin** |  |  | 0.1241 | 1.132 | 0.534, 2.401 | 0.7462 |
| **Joint Association** |  |  | -0.1349 | 0.874 | 0.327, 2.335 | 0.7878 |
| **Ages 35-44** | 21/2,121 |  |  |  |  |  |
| **Triglycerides** |  |  | 0.3489 | 1.418 | 0.693, 2.900 | 0.3395 |
| **CRP** |  |  | 0.4521 | 1.572 | 0.648, 3.814 | 0.3175 |
| **Glycohemoglobin** |  |  | -0.0889 | 0.915 | 0.731, 1.145 | 0.4375 |
| **Joint Association** |  |  | 0.7121 | 2.038 | 0.868, 4.787 | 0.1021 |
| **Ages 45-60** | 81/3,163 |  |  |  |  |  |
| **Triglycerides** |  |  | 0.1559 | 1.169 | 0.837, 1.632 | 0.3598 |
| **CRP** |  |  | -0.0294 | 0.971 | 0.589, 1.600 | 0.9080 |
| **Glycohemoglobin** |  |  | 0.1343 | 1.144 | 1.023, 1.279 | 0.0188 |
| **Joint Association** |  |  | 0.2608 | 1.298 | 0.726, 2.321 | 0.3793 |
| **Ages 65+** | 307/3,944 |  |  |  |  |  |
| **Triglycerides** |  |  | 0.0143 | 1.014 | 0.803, 1.282 | 0.9049 |
| **CRP** |  |  | 0.3688 | 1.446 | 1.074, 1.947 | 0.0151 |
| **Glycohemoglobin** |  |  | 0.0543 | 1.056 | 0.969, 1.150 | 0.2124 |
| **Joint Association** |  |  | 0.4373 | 1.549 | 1.101, 2.178 | 0.0119 |
| ^a^Models adjusted for age, gender, race/ethnicity, BMI, smoking status, and family income.  ^b^OR estimates calculated for an IQR change in the respective biomarker, or an IQR in each  biomarker for the joint association estimates. IQRs for ln(Triglycerides, mg/dL) = 0.798 [corresponding to a 2.22 fold increase in non-transformed Triglycerides], ln(CRP, mg/dL) = 2.22 [corresponding to a 9.21 fold increase in non-transformed CRP], ln(Glycohemoglobin, %) = 0.0935 [corresponding to a 1.10 fold increase in non-transformed glycohemoglobin]. | | | | | | |

| **Supplemental Table 4e. Age-Stratified Adjusted^a^ Log-Transformed Joint Association Logistic Regression Model Results - Coronary Heart Disease** | | | | | | |
| --- | --- | --- | --- | --- | --- | --- |
|  | **cases/*n*** |  | **β*IQR** | **OR^b^** | **(95% CI)** | **p-value** |
| **Coronary Heart Disease** | | | | | | |
| **Ages 20-34^c^** | 3/3,321 |  |  |  |  |  |
| **Triglycerides** |  |  | --- | --- | --- | --- |
| **CRP** |  |  | --- | --- | --- | --- |
| **Glycohemoglobin** |  |  | --- | --- | --- | --- |
| **Joint Association** |  |  | --- | --- | --- | --- |
| **Ages 35-44** | 13/2,121 |  |  |  |  |  |
| **Triglycerides** |  |  | 0.3119 | 1.366 | 0.506, 3.687 | 0.5382 |
| **CRP** |  |  | 1.0951 | 2.989 | 1.026, 8.714 | 0.0448 |
| **Glycohemoglobin** |  |  | 0.0610 | 1.063 | 0.854, 1.323 | 0.5846 |
| **Joint Association** |  |  | 1.4679 | 4.340 | 1.289, 14.618 | 0.0178 |
| **Ages 45-60** | 99/3,157 |  |  |  |  |  |
| **Triglycerides** |  |  | 0.1812 | 1.199 | 0.946, 1.518 | 0.1332 |
| **CRP** |  |  | -0.1468 | 0.864 | 0.470, 1.588 | 0.6367 |
| **Glycohemoglobin** |  |  | 0.1875 | 1.206 | 1.057, 1.377 | 0.0056 |
| **Joint Association** |  |  | 0.2218 | 1.248 | 0.689, 2.261 | 0.4641 |
| **Ages 65+** | 393/3,909 |  |  |  |  |  |
| **Triglycerides** |  |  | 0.0773 | 1.080 | 0.897, 1.301 | 0.4153 |
| **CRP** |  |  | -0.0708 | 0.932 | 0.684, 1.268 | 0.6529 |
| **Glycohemoglobin** |  |  | 0.1372 | 1.147 | 1.061, 1.240 | 0.0006 |
| **Joint Association** |  |  | 0.1437 | 1.155 | 0.811, 1.644 | 0.4255 |
| ^a^Models adjusted for age, gender, race/ethnicity, BMI, smoking status, and family income.  ^b^OR estimates calculated for an IQR change in the respective biomarker, or an IQR in each  biomarker for the joint association estimates. IQRs for ln(Triglycerides, mg/dL) = 0.798 [corresponding to a 2.22 fold increase in non-transformed Triglycerides], ln(CRP, mg/dL) = 2.22 [corresponding to a 9.21 fold increase in non-transformed CRP], ln(Glycohemoglobin, %) = 0.0935 [corresponding to a 1.10 fold increase in non-transformed glycohemoglobin].  ^c^Model convergence criteria not satisfied. | | | | | | |

1. **Third Report of the National Cholesterol Education Program (NCEP) Expert Panel on Detection, Evaluation, and Treatment of High Blood Cholesterol in Adults (Adult Treatment Panel III) final report**. *Circulation* 2002, **106**(25):3143-3421. [↑](#footnote-ref-1)
